# Discovering recent selection forces shaping the evolution of dengue viruses based on polymorphism data across geographic scales

**DOI:** 10.1093/ve/veac108

**Published:** 2022-11-29

**Authors:** Nien-Kung Li, Jukka Corander, Yonatan H Grad, Hsiao-Han Chang

**Affiliations:** Department of Life Science & Institute of Bioinformatics and Structural Biology, National Tsing Hua University, 101, Section 2, Kuang-Fu Road, Hsinchu 300044, Taiwan; Helsinki Institute for Information Technology, Department of Mathematics and Statistics, University of Helsinki, Yliopistonkatu 3, Helsinki 00014, Finland; Department of Biostatistics, University of Oslo, Domus Medica Gaustad Sognsvannsveien 9, Oslo 0372, Norway; Parasites and Microbes, The Wellcome Sanger Institute, Wellcome Trust Genome Campus, Hinxton, Cambridge CB10 1SA, UK; Department of Immunology and Infectious Diseases and Center for Communicable Disease Dynamics, Harvard T. H. Chan School of Public Health, 677 Huntington Ave, Boston, Massachusetts 02115, USA; Division of Infectious Diseases, Brigham and Women’s Hospital, Harvard Medical School, 75 Francis St, Boston, Massachusetts 02115, USA

**Keywords:** dn/ds, pMK test, natural selection, dengue virus, geographic scales

## Abstract

Incomplete selection makes it challenging to infer selection on genes at short time scales, especially for microorganisms, due to stronger linkage between loci. However, in many cases, the selective force changes with environment, time, or other factors, and it is of great interest to understand selective forces at this level to answer relevant biological questions. We developed a new method that uses the change in *d_N_*/*d_S_*, instead of the absolute value of *d_N_*/*d_S_*, to infer the dominating selective force based on sequence data across geographical scales. If a gene was under positive selection, *d_N_*/*d_S_* was expected to increase through time, whereas if a gene was under negative selection, *d_N_*/*d_S_* was expected to decrease through time. Assuming that the migration rate decreased and the divergence time between samples increased from between-continent, within-continent different-country, to within-country level, *d_N_*/*d_S_* of a gene dominated by positive selection was expected to increase with increasing geographical scales, and the opposite trend was expected in the case of negative selection. Motivated by the McDonald–Kreitman (MK) test, we developed a pairwise MK test to assess the statistical significance of detected trends in *d_N_*/*d_S_*. Application of the method to a global sample of dengue virus genomes identified multiple significant signatures of selection in both the structural and non-structural proteins. Because this method does not require allele frequency estimates and uses synonymous mutations for comparison, it is less prone to sampling error, providing a way to infer selection forces within species using publicly available genomic data from locations over broad geographical scales.

## Introduction

With the decrease in sequencing costs, genome sequences of representatives of a species across geographic and temporal scales are increasingly available (Gratton et al. 2017), providing opportunities to understand the species’ recent demographic history and evolution. The ratio of non-synonymous to synonymous substitution rates (*d_N_*/*d_S_*), which compares the relative abundance of amino acid altering and preserving mutations, is commonly used to infer selection between species. Because *d_N_*/*d_S_* is applicable even when recombination is absent (Hedge, Wilson, and Ouellette 2016), it is useful for haploid organisms, such as bacteria and viruses, in which tests based on linkage disequilibrium cannot be applied (Shapiro et al. 2009). Traditionally, a *d_N_*/*d_S_* larger than one is considered a signal of positive selection, a *d_N_*/*d_S_* smaller than one is considered a signal of negative selection, and *d_N_*/*d_S_* is expected to be one under a neutral condition. However, at the within-species level, since the divergence time among samples is shorter, selection may not have had sufficient time to act within the sampling timeframe, leading to incomplete selection and possible misinterpretation (Rocha et al. 2006; Kryazhimskiy, Plotkin, and Gojobori 2008; Mugal, Wolf, and Kaj 2014). For example, incomplete purifying selection may lead to elevated *d_N_*/*d_S_* (Hasegawa, Cao, and Yang 1998; Pybus et al. 2007; Peterson and Masel 2009; Park et al. 2015).

Since within-species *d_N_*/*d_S_* should be interpreted with caution, several studies formally incorporated polymorphisms in their models when estimating *d_N_*/*d_S_* (Wilson et al. 2011; Mugal et al. 2020; Wilson and Consortium 2020), with the assumption that selection did not change over space and time. Bhatt et al. considered that high-frequency non-synonymous polymorphic sites are possibly adaptive and utilized segregating mutations with different frequencies to better infer the rate of adaptation (Bhatt, Katzourakis, and Pybus 2010; Bhatt, Holmes, and Pybus 2011). However, since this method requires estimates of allele frequencies, it is sensitive to sampling bias and, therefore, less suitable for analyzing samples in public databases, which often represent datasets collected and sequenced by multiple research groups. Moreover, these models also assume a constant force of selection, an assumption that may be violated in the case of local adaptation.

Since *d_N_*/*d_S_* of a positively selected gene is expected to increase through time and *d_N_*/*d_S_* of a negatively selected gene is expected to decrease through time (Mugal, Wolf, and Kaj 2014), examining the trend of change in *d_N_*/*d_S_* can provide information about recent selective forces. The straightforward way to observe the change in *d_N_*/*d_S_* is to analyze temporal samples. Alternatively, samples across geographic scales may represent different levels of divergence time. It has been shown in several organisms that the genetic differentiation, which decreases with gene flow or migration rate between populations, was higher between continents than within each continent (Romualdi et al. 2002; Bedford et al. 2010; Yukilevich et al. 2010; Miotto et al. 2013; Azarian et al. 2018). Suppose we assume that gene flow decreases with geographic distances (e.g. within-continent gene flow is higher than between-continent gene flow, and within-country gene flow is higher than between-country gene flow) and since divergent time is expected to decrease with gene flow, divergent time between samples increases with geographic distances. In other words, analyzing samples from locations across geographic scales provides an opportunity to obtain *d_N_*/*d_S_* at different levels of divergence time.

We applied this idea to study recent selective forces acting on four serotypes of dengue viruses, which have been shown to emerge from four sylvatic ancestors independently (Holmes and Twiddy 2003) and differ in their virulence (Fried et al. 2010) and transmissibility (Duong et al. 2015). Sequence data of dengue viruses across geographic scales are publicly available, offering a great opportunity to study the evolution of this important pathogen. The statistical significance was tested using a simple contingency test adapted from the original McDonald–Kreitman (MK) test (McDonald and Kreitman 1991). Across dengue genes, we found that the dominant selective force varied among serotypes and continents, providing evolutionary insights into the phenotypic difference among serotypes.

## Materials and methods

### Genomic data of dengue viruses

We downloaded all the available sequences of four dengue virus serotypes (DENV1–4) from the National Center for Biotechnology Information database on 11 February 2020 (NCBI 1988). After excluding sequences from non-human sources, the sample sizes for serotypes 1, 2, 3, and 4 are 1,150, 865, 707, and 198 from 31, 33, 38, and 22 countries, respectively. Bayesian clustering was performed using the R package *rhierBAPS* (Tonkin-Hill et al. 2018) (*max.depth* = 3).

### *d_N_*/*d_S_* estimation and pairwise McDonald–Kreitman test

The ratio of non-synonymous to synonymous changes (*d_N_*/*d_S_*) was calculated between every pair of sequences using the maximum likelihood method implemented in CodeML from *PAML* (Yang 1997, 2007) (runmode = −2, CodonFreq = 2), and the average *d_N_*/*d_S_* was calculated by taking the ratio of the average *d_N_* to the average *d_S_*. The number of non-synonymous changes per non-synonymous site, *d_N_*, is equal to *C*_N_/*N*, where *C*_N_ is the number of non-synonymous changes and *N* is the total number of non-synonymous sites. Similarly, the number of synonymous changes per synonymous site, *d_S_*, is equal to *C*_S_/*S*, where *C*_S_ is the number of synonymous changes and *S* is the total number of synonymous sites.

To examine the statistical significance of the pattern of *d_N_*/*d_S_* across geographic scales, we modified the original MK test to what we term a pairwise McDonald–Kreitman (pMK) test. The numbers of synonymous and non-synonymous changes, *C_S_* and *C_N_*, were calculated from the numbers of non-synonymous and synonymous sites (*N* and *S*), and *d_N_* and *d_S_* were estimated from *PAML* by *N* × *d_N_* and *S* × *d_S_*, respectively.

We compared the non-synonymous and synonymous differences between within-continent between-country and between-continent levels. We first calculated the average pairwise non-synonymous and synonymous differences between all the country pairs and then summed and rounded the differences between continents or within each continent. The two dimensions of the contingency test were (1) within or between continents and (2) non-synonymous or synonymous (Table 1). For within-continent changes, we used either the America or Asia data. Additionally, to test the robustness of the results, we performed the same tests with only values within the interquartile range (pMK* test). We estimated *q*-values using the *qvalue* package (Storey et al. 2020) in R and used *q *< 0.1 as a standard for statistical significance. To quantify the magnitude of the signal, we also calculated the odds ratio as follows:

**Table 1.** The components of the pMK test.

	Between continents	Within continent
Non-synonymous	A	C
Synonymous	B	D

$$\frac{{{\raise0.7ex\hbox{$A$} \!\mathord{\left/{\vphantom {A B}}\right.}
\!\lower0.7ex\hbox{$B$}}}}{{{\raise0.7ex\hbox{$C$} \!\mathord{\left/{\vphantom {C D}}\right.}\!\lower0.7ex\hbox{$D$}}}}.$$

The difference in divergence times, *S* ratio, was quantified by the ratio of synonymous changes between and within continents, *B*/*D*.

### Simulations

We used SLiM to simulate positive and negative selection with various values of selection coefficients (*s* = 0.0025, 0.005, 0.01, and 0.02) and migration rates (5 × 10^−6^, 5 × 10^−5^, 5 × 10^−4^, 5 × 10^−3^, and 5 × 10^−2^) (Haller, Messer, and Hernandez 2019). Haploid populations with no recombination event were simulated. The population size was 1,000 per subpopulation, and the mutation rates were 3 × 10^−5^, 1 × 10^−5^, and 1 × 10^−6^ for neutral, deleterious, and beneficial mutations, respectively. We considered ten pairs of locations in simulations, which is analogous to five countries per continent. Each simulation scenario was repeated 100 times, and the average was presented.

## Results

**Figure 1.**
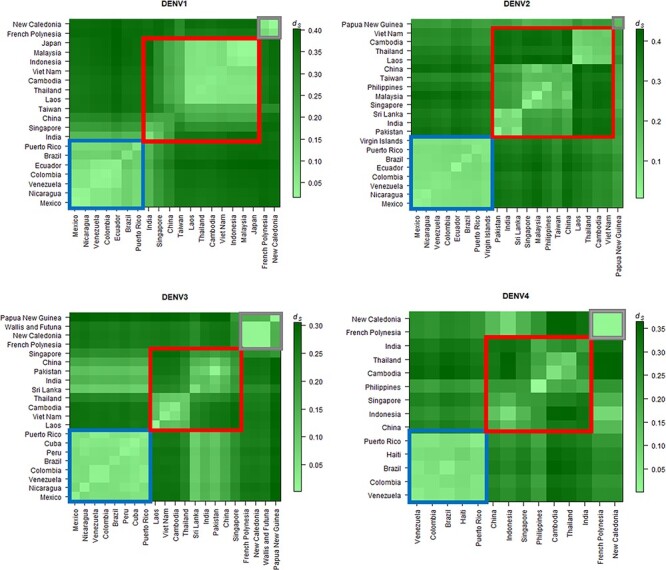
Population substructure of dengue viruses. The population substructure of dengue viruses was characterized by the average synonymous substitution rates (*d_S_*) between each pair of countries. Between-continent differentiation was higher than within-continent differentiation. Countries in Asia, the Americas, and Oceania are indicated by red, blue, and grey borders, respectively. For DENV2 and DENV3, Asian countries were separated into two subgroups (DENV2 group 1 includes Laos, Thailand, Cambodia, and Vietnam; DENV2 group 2 includes Pakistan, India, Sri Lanka, Singapore, Malaysia, Philippines, Taiwan, and China; DENV3 group 1 includes Laos, Vietnam, Cambodia, Thailand, and Singapore; DENV3 group 2 includes Sri Lanka, India, Pakistan, and China).

We developed and applied the new method based on the change in *d_N_*/*d_S_* to the dengue virus to understand recent selection in all four serotypes. First, to know if the samples showed multiple levels of divergence time, we examined the population structure of dengue viruses using two approaches: (1) synonymous changes and (2) a Bayesian clustering method. Assuming that synonymous changes are neutral, *d_S_* is expected to increase with the divergence time between samples (Nei and Kumar 2000) and therefore was used to reveal relative divergence time across geographic scales. The Bayesian clustering method, *rhierBAPS*, assigns samples that are genetically more similar to the same groups. Results from both approaches suggest a clear population structure shaped by continental differences (Fig. 1 and Supplementary Fig. S1). Varying levels of substructure in Asia were found among serotypes by pairwise *d_S_*, and the divergence among countries in the Americas was lower than that among countries in Asia for all the serotypes (Fig. 1). In addition, groups identified using a Bayesian clustering method (called as ‘BAPS groups’) tended to be composed of samples from the same continents (Supplementary Fig. S1), revealing substantial genetic differentiation between continents.

**Figure 2.**
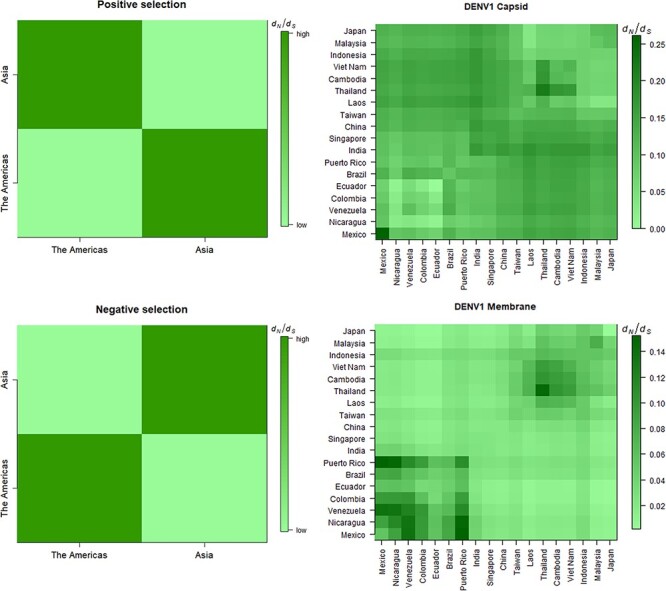
The patterns of *d_N_*_/_*d_S_* under positive and negative selection. For genes dominated by positive selection, we expected larger between-continent *d_N_*_/_*d_S_*; for genes dominated by negative selection, we expected larger within-continent *d_N_*_/_*d_S_*. For each of the two scenarios, an empirical example is shown on the right.

Since both synonymous changes and Bayesian clustering suggest that gene flow between continents was significantly lower than that within continents, we examined the changes in average *d_N_*/*d_S_* and inferred the dominant selective force using samples covering broad geographic scales and representing a wide range of divergence times. Since DENV2 and DENV3 showed a population substructure within Asia, we separated Asian countries into two subgroups for the following analysis (Fig. 1). To obtain the average *d_N_*/*d_S_* for each pair of countries, we calculated the averages of *d_N_* and *d_S_* over all pairs of samples and then took the ratio. For genes dominated by positive selection, the average *d_N_*/*d_S_* should increase with the increasing geographical distance and the opposite was expected for genes dominated by negative selection (Fig. 2). It is also possible that the selective force varied between continents (Supplementary Fig. S2)—for example, it could appear only in one continent or differ between two continents.

**Figure 3.**
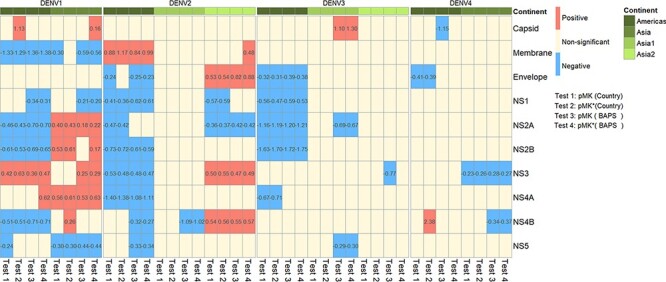
The summarized results of the pMK test. The figure summarizes the results of the pMK test for ten genes across all four serotypes and two continents (the Americas, Asia, Asia group 1, and Asia group 2). The color of each box indicates positive selection (red), negative selection (blue), or non-significant results (ivory); the log odds ratios of significant results are shown in the box. In Test 1 and Test 2, the continent of a sample was determined by its geographic location (labeled by ‘country’). In Test 3 and Test 4, the continent of a sample was determined by the geographic origin of the majority of samples within a BAPS group (labeled by ‘BAPS’). In Test 1 and Test 3, all the values were used (pMK test); in Test 2 and Test 4, only values within the interquartile range were used (pMK* test). Selective forces varied between serotypes (in the capsid, membrane, NS3, and NS4A genes) and continents (envelope, NS2A, NS2B, and NS3). The results from Test 1 to Test 4 were consistent, while the number of significant results differed.

To further determine the statistical significance of changing *d_N_*/*d_S_*, we developed a pMK test (Supplementary Fig. S3). The first dimension of the contingency table (Table 1) is synonymous or non-synonymous, and the second dimension is within or between continents. Compared to synonymous changes, more non-synonymous changes between continents than within continents were expected in a gene dominated by positive selection and the opposite applied to negative selection. With the contingency table (Table 1), we calculated the log odds ratio $\left( {\frac{{{\raise0.7ex\hbox{$A$} \!\mathord{\left/{\vphantom {A B}}\right.}\!\lower0.7ex\hbox{$B$}}}}{{{\raise0.7ex\hbox{$C$} \!\mathord{\left/{\vphantom {C D}}\right.} \!\lower0.7ex\hbox{$D$}}}}} \right)$ to quantify the intensity of the signal and the *S* ratio (${\raise0.7ex\hbox{$B$} \!\mathord{\left/{\vphantom {B D}}\right.}
\!\lower0.7ex\hbox{$D$}}$) to quantify the difference in the divergence time. The median log *S* ratio was 1.46 (DENV1), 1.59 (DENV2), 1.55 (DENV3), and 1.45 (DENV4) for the Americas,; 0.46 (DENV1) and 0.01 (DENV4) for Asia; 1.15 (DENV2) and 0.45 (DENV3) for Asia group 1; and 0.65 (DENV2) and 0.57 (DENV3) for Asia group 2, suggesting a higher difference in divergence times when the pMK test was applied to the Americas. Because geographic locations of imported cases did not reflect where the infection occurred and could potentially influence our analysis, we determined ‘continents’ in two ways: first, by each sample’s geographic location and second by the geographic origin of the majority of samples within a group identified by *rhierBAPS* (Fig. 3 and Supplementary Fig. S4).

We found that selective forces varied between serotypes (in the capsid, membrane, NS3, and NS4A genes) and continents (envelope, NS2A, NS2B, and NS3) (Fig. 3). The number of genes showing evidence of positive selection was higher in Asia than in the Americas. The results from the tests based on country grouping and BAPS grouping were consistent, with the latter providing more significant results (59 [country] vs 73 [BAPS]). To examine the impact of outliers, we also performed the test with only values within the interquartile range (pMK* test). The results generally remained similar, with some differences in statistical significance (5 [country] or 7 [BAPS] out of 100 pairs of tests) but no difference in the type of selection. The intensity of the signal ranged from 0.16 to 2.38 for positive selection and from −1.75 to −0.20 for negative selection, with the capsid of DENV1 and DENV3 and the membrane of DENV2 showing the greatest signal of positive selection and NS2B of DENV3 showing the strongest signal of negative selection (Fig. 3).

We further characterized the selective force for each domain of the two genes with known structures (Modis et al. 2004, 2005; Nayak et al. 2009; Hertz et al. 2017), the envelope protein, and NS1 (Supplementary Fig. S4 and Table S1). While NS1 was found to be under purifying selection if the whole gene was analyzed, evidence of positive selection was found in Domain 2, suggesting varying selection forces among different functional domains. A similar pattern of varying selective force across domains was also found in the envelope protein. For example, for Serotype 3 in the Americas, the whole gene and Domain 2 showed signals of negative selection, while Domain 1 was shown to be under positive selection.

**Figure 4.**
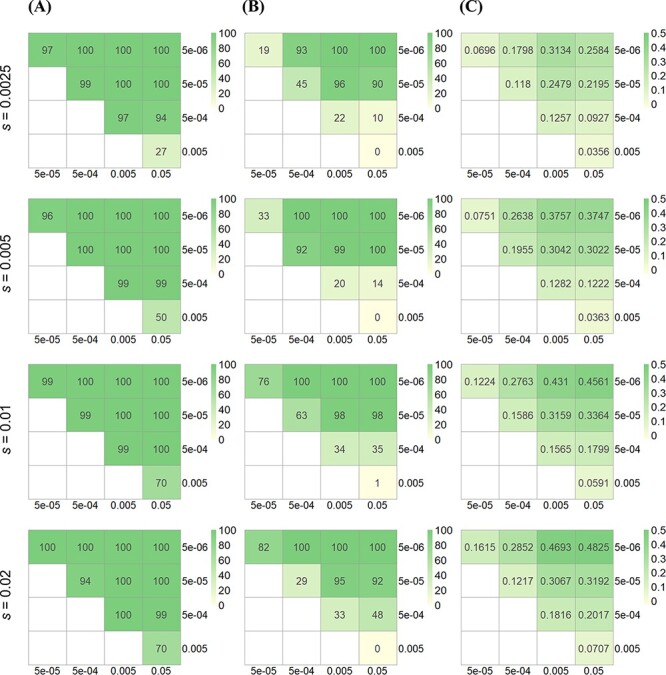
Simulations showed that the power of the pMK test varied with migration rates and selection coefficients (positive selection). The power of the pMK test across different levels of divergence time was examined through simulating various migration rates. Higher migration rates represent lower divergence times. (A) The number in each box indicates the number of replicates showing the expected pattern of positive selection (i.e. *A*/*B* > *C*/*D*) out of 100 replicates. The majority of replicates showed expected patterns, and the consistency was the lowest when migration rates were both high. (B) The number in each box indicates the number of replicates with significant pMK test results (p-value <0.05) out of 100 replicates. The number of significant replicates increased with the difference in migration rates and the selection coefficient. (C) The number in each box represents the average log odds ratio (i.e. log$\left( {\frac{{{\raise0.7ex\hbox{$A$} \!\mathord{\left/{\vphantom {A B}}\right.} \!\lower0.7ex\hbox{$B$}}}}{{{\raise0.7ex\hbox{$C$} \!\mathord{\left/{\vphantom {C D}}\right.}\!\lower0.7ex\hbox{$D$}}}}} \right)$). The average log odds ratio increased when the selection coefficient and the difference in migration rates increased.

Finally, we performed simulations to explore how the variation of migration rates between populations and selection intensity influenced the power of the pMK test and the magnitude of the selection signal (Fig. 4 and Supplementary Fig. S5). As expected, the power of the pMK test and the intensity of the signal increased with the strength of selection. Moreover, the larger the difference in migration rates between populations, or say, the larger the difference in divergence times, the higher the power of the pMK test and the magnitude of the selection signal.

When the log *S* ratio, which represents the difference in divergence times, was greater than 1 (Supplementary Fig. S6), positive selection with a selection coefficient greater than 0.0025 and negative selection with a selection coefficient smaller than −0.01 can generally be detected (Supplementary Fig. S6). Since the magnitude of the signal was influenced by both the selection coefficient and the difference in migration rates (Fig. 4C and Supplementary Fig. S5C), the log odds ratio cannot be used to infer the strength of selection directly; however, under the same set of migration rates, the log odds ratio can be used to compare the relative strength of selection between genes.

## Discussion and conclusion

We developed a new approach based on the change in *d_N_*/*d_S_* to infer the dominating selective force using within-species polymorphism data. Because selective constraints likely influence most sites in most genes, if considering a gene as a whole, *d_N_*/*d_S_* is rarely greater than 1. However, some particular sites in a gene may be under positive selection, and using the threshold of 1 to identify positive selection can potentially overlook interesting and important biological observations. Since our method does not require *d_N_*/*d_S_* to be greater than 1 for positive selection, it better captures these signals. In fact, none of the genes showed average *d_N_*/*d_S_* greater than 1 in our analysis. Moreover, using the average pairwise *d_N_*/*d_S_* across geographic scales in the pMK test, our method considers all the polymorphic differences, not just the fixed differences between locations or species that is used in the MK test (e.g. Parsch, Zhang, and Baines 2009; Fay 2011), increasing the chance of capturing recent selective events. However, it is important to note that, even if a gene is inferred to be ‘dominated’ by positive selection, some sites (and likely the majority) may still be under purifying selection.

Our analysis did not assume a single value of *d_N_*/*d_S_* across all geographic locations, nor did we assume the same selective force throughout the gene. However, similar to other studies using synonymous sites as a control (Spielman and Wilke 2015), we did assume that synonymous mutations are neutral. Thus, if synonymous sites are under selection, it can lead to false-positive or false-negative results. Potentially, comparing the change in *d_S_* between genes across geographic scales can provide insights into selection on synonymous sites and help interpret the overall results of *d_N_*/*d_S_*. Moreover, we also assumed that, compared to the difference in divergence time between samples from different continents, the difference in sampling time is relatively small.

Through this new approach, we identified dominant selective forces acting on the evolution of four dengue serotypes. We found signatures of positive selection in the envelope proteins, NS2A, NS2B, and NS4B, which have been reported in previous studies (Twiddy et al. 2002a; Twiddy, Woelk, and Holmes 2002b; Bennett et al. 2003; Lin et al. 2019). Additionally, we found that positive selection also acted on the evolution of capsid, membrane, NS3, and NS4A in at least one region and one serotype. By comparing our results with a previous study based on the site model (Twiddy, Woelk, and Holmes 2002b), we found that our approach was able to capture signals of positive selection for a gene even when only a small proportion of sites were under positive selection (e.g. envelope of DENV2). Moreover, while NS1 was shown to be under purifying selection in our study and previous studies (Twiddy, Woelk, and Holmes 2002b; Lin et al. 2019), we performed additional analysis for each domain separately and identified domains of NS1 influenced by positive selection. Finally, we found that the dominant selective forces inferred in this study differed between continents in some of the genes, and the reason behind this remains to be explored. Since dengue viruses became widespread in the Americas later than in Asia, it is possible that (1) selective pressures imposed by human immune responses differed between continents due to the difference in the proportion of the population that had been infected by dengue viruses before or (2) the recent bottleneck decreased the power to detect positive selection in the Americas (Parsch, Zhang, and Baines 2009).

While we applied our method to an organism with a clear population structure, it can also be used for organisms where the population structure is either more continuous or less obvious. If there is a clear pattern of isolation by distance, a correlation test or segmented linear regression between *d_N_*/*d_S_* and the geographical distance can potentially be used to infer dominant selective forces. A similar concept can be applied to temporal data when sufficient genomic data from multiple time points are available.

In summary, this study presents a new and simple method to detect selection at the within-species level. Since publicly available genomic data from locations over broad geographical scales are more common than temporal data, our idea of using samples across geographic scales to represent a range of divergence time opens up opportunities for more organisms. Our results suggest that dominant selective forces varied among serotypes and continents in dengue viruses and provide insights into the evolution and biology of dengue viruses and candidate regions that may warrant further investigation.

## Supplementary Material

veac108_Supp

## Data Availability

The sequences used in this study and the example code for the pMK test can be downloaded from our GitHub repository (https://github.com/hhc-lab/dengue_selection).
