# Supplementary material for: Discovering recent selection forces shaping the evolution of dengue viruses based on polymorphism data across geographic scales
Source: Virus Evol. 2022 Nov 29;8(2):veac108. doi: 10.1093/ve/veac108 (PMC9789396; doi:10.1093/ve/veac108)
Supplement: veac108_Supp [file veac108_supp.zip › suppl_data/supplementary_materials_revised_1128.pdf]

Supplementary Materials

Supplementary text

Genomic data processing

We used the iterative refinement method FFT-NS-i in MAFFT (Kuraku et al. 2013; Katoh et al. 2019) version 7 to align the sequences. Recombination blocks were detected using recombination detection program version 4 (RDP4) (Martin et al. 2015) and replaced by gaps (-). For each gene, samples with more than 5% gaps were removed from the analysis.

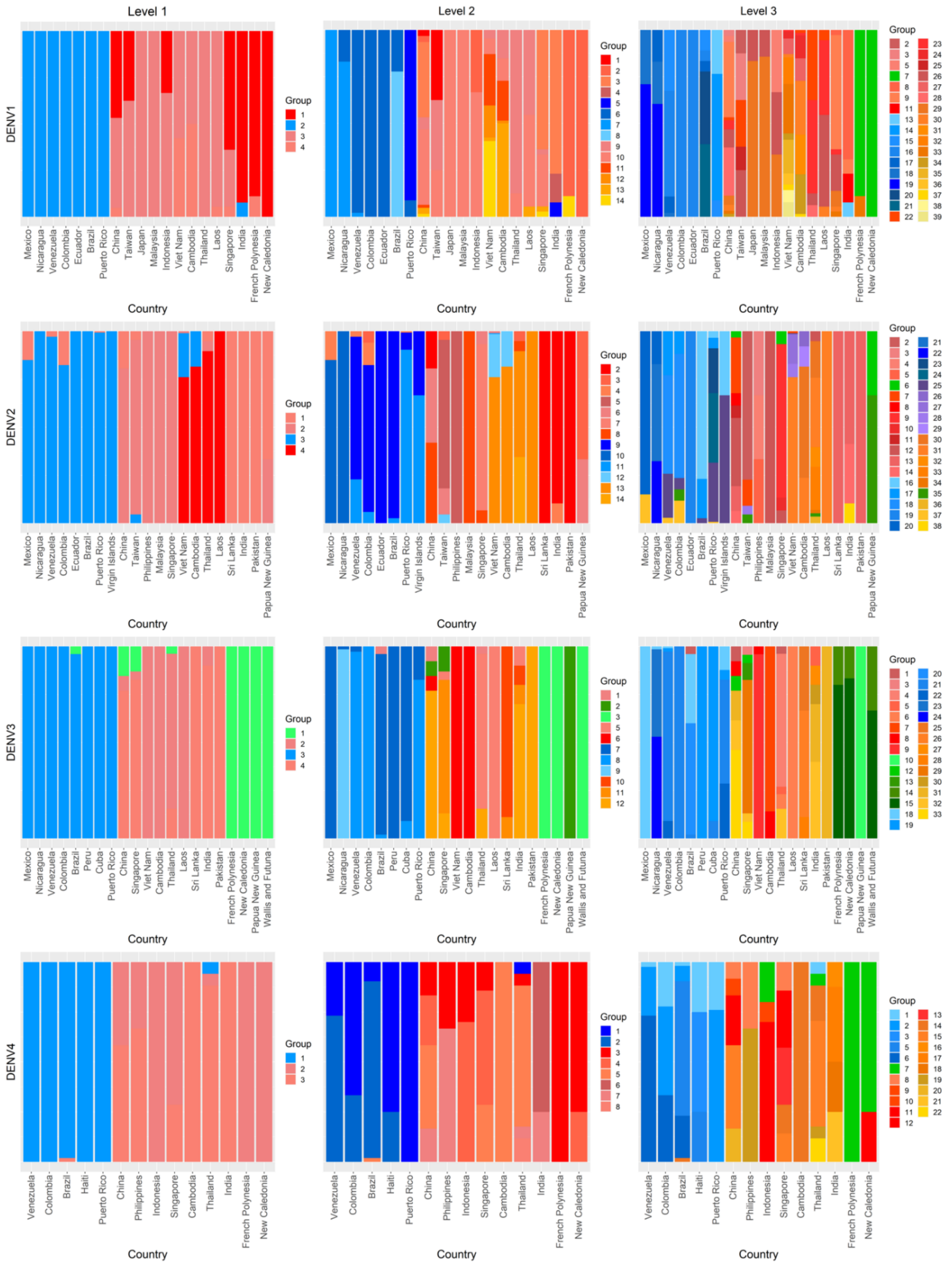

Figure S1. Population substructure revealed by BAPS clustering. Three levels of clustering are shown. The groups within which most countries were from the Americas were labeled by blue or purple colors; the groups within which most countries were from Asia were labeled by red, orange, or yellow colors; other groups were labeled by green colors.

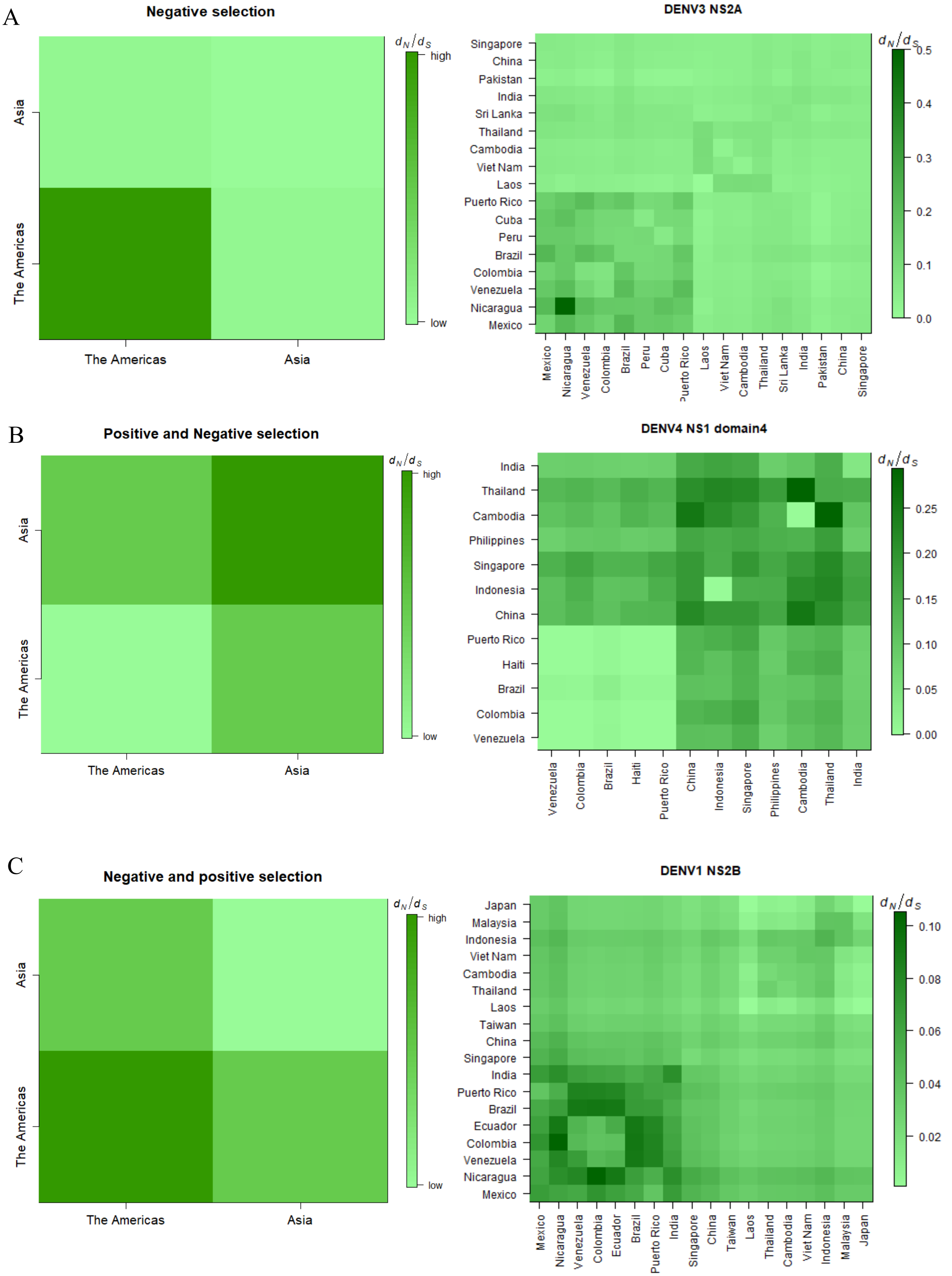

A
Negative selection
DENV3 NS2A
B
Positive and Negative selection
DENV4 NS1 domain4
C
Negative and positive selection
DENV1 NS2B
The Americas
Asia
high
low
Singapore
China
Pakistan
India
Sri Lanka
Thailand
Cambodia
Viet Nam
Laos
Puerto Rico
Cuba
Peru
Brazil
Colombia
Venezuela
Nicaragua
Mexico
Philippines
Indonesia
Haiti
Japan
Malaysia
Taiwan
Ecuador
0.0
0.1
0.2
0.3
0.4
0.5
0.00
0.05
0.10
0.15
0.20
0.25
0.02
0.04
0.06
0.08
0.10

Figure S2. Other types of patterns of d_N / d_S within and between continents. (A) The changing pattern of d_N / d_S is significant only when comparing within-continent d_N / d_S from one continent (in this case, the Americas) with between-continent d_N / d_S. (B)(C) The changing pattern of d_N / d_S is significant for both continents, but the inferred dominant selective differs.

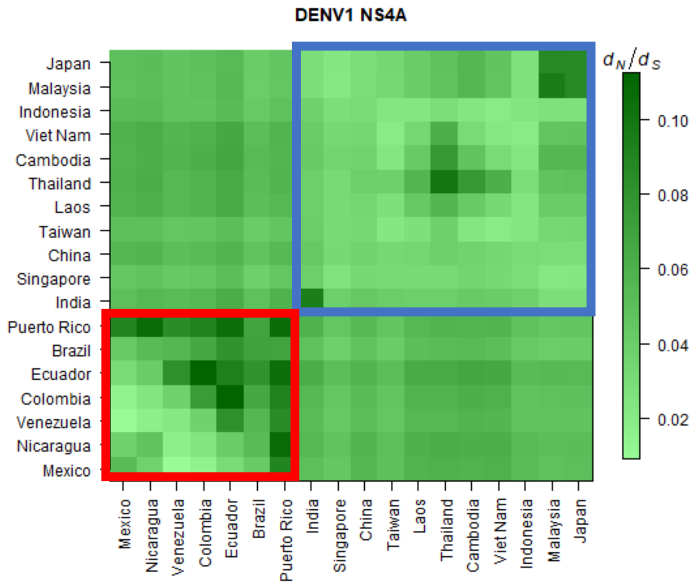

	Between continents	Within Asia
**Nonsynonymous**	429	91
**Synonymous**	4286	1584
**Ratio**	0.100	0.057
***P*-value**		1.20×10^{-6}
***q*-value**		1.21×10^{-5}

	Between continents	Within the Americas
**Nonsynonymous**	386	16
**Synonymous**	3924	222
**Ratio**	0.098	0.072
***P*-value**		0.29
***q*-value**		0.56

Figure S3. An example of the pMK test. In this example, the nonsynonymous-to-synonymous ratio within Asia was significantly smaller than that between continents and a force of positive selection was inferred. The result for the Americas was not significant.

(A)

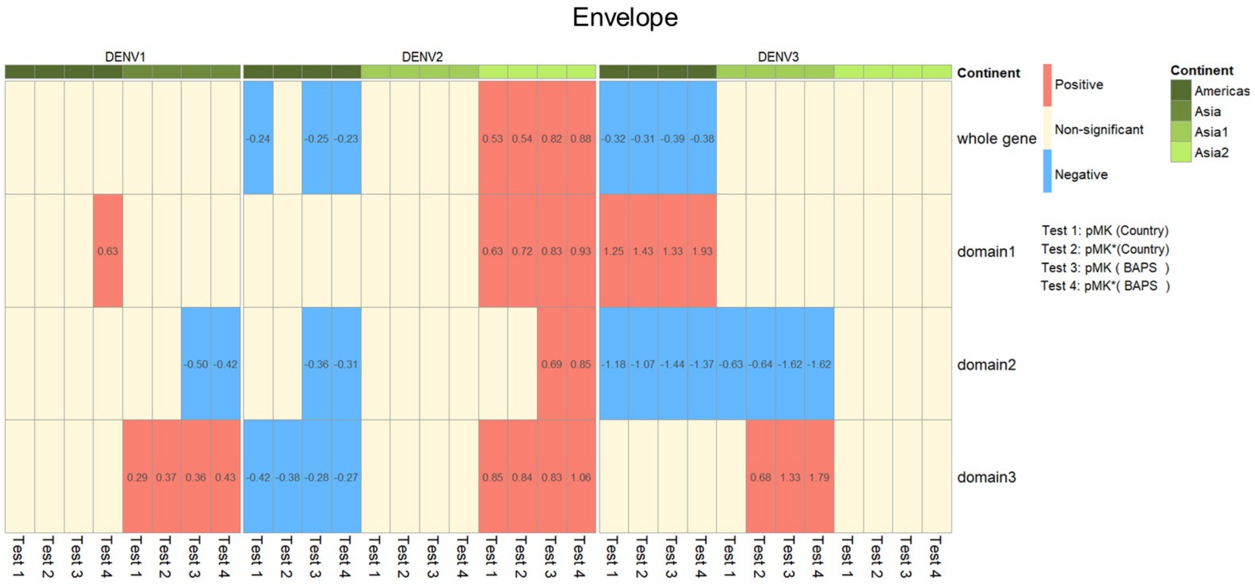

(B)

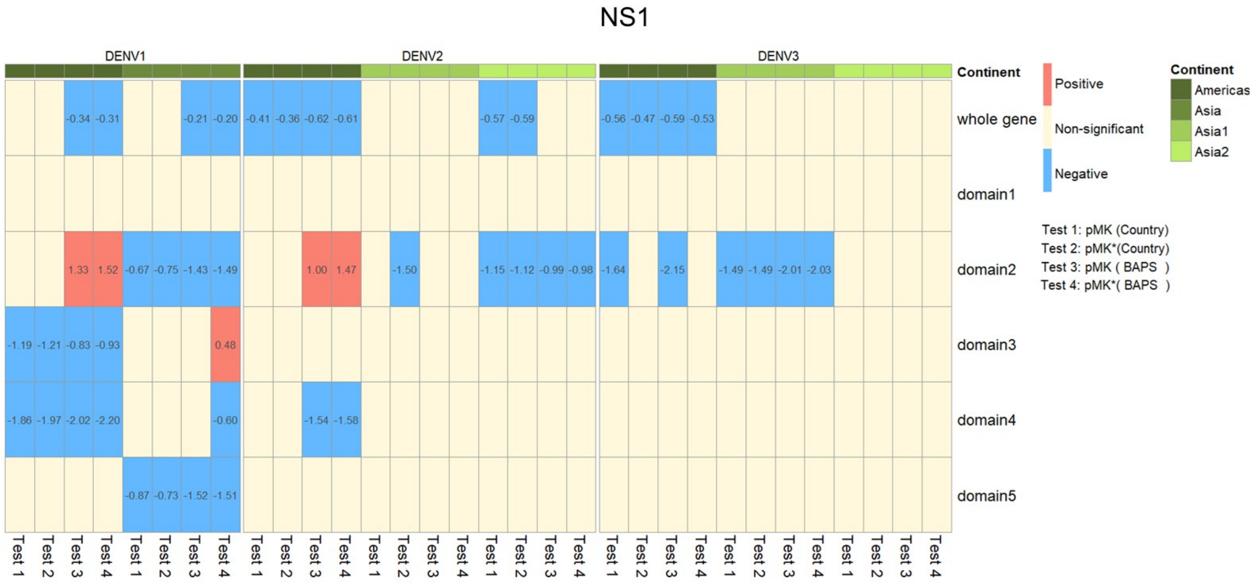

Figure S4. The summarized results of the pMK test for different domains of the envelope protein (A) and NS1 (B).

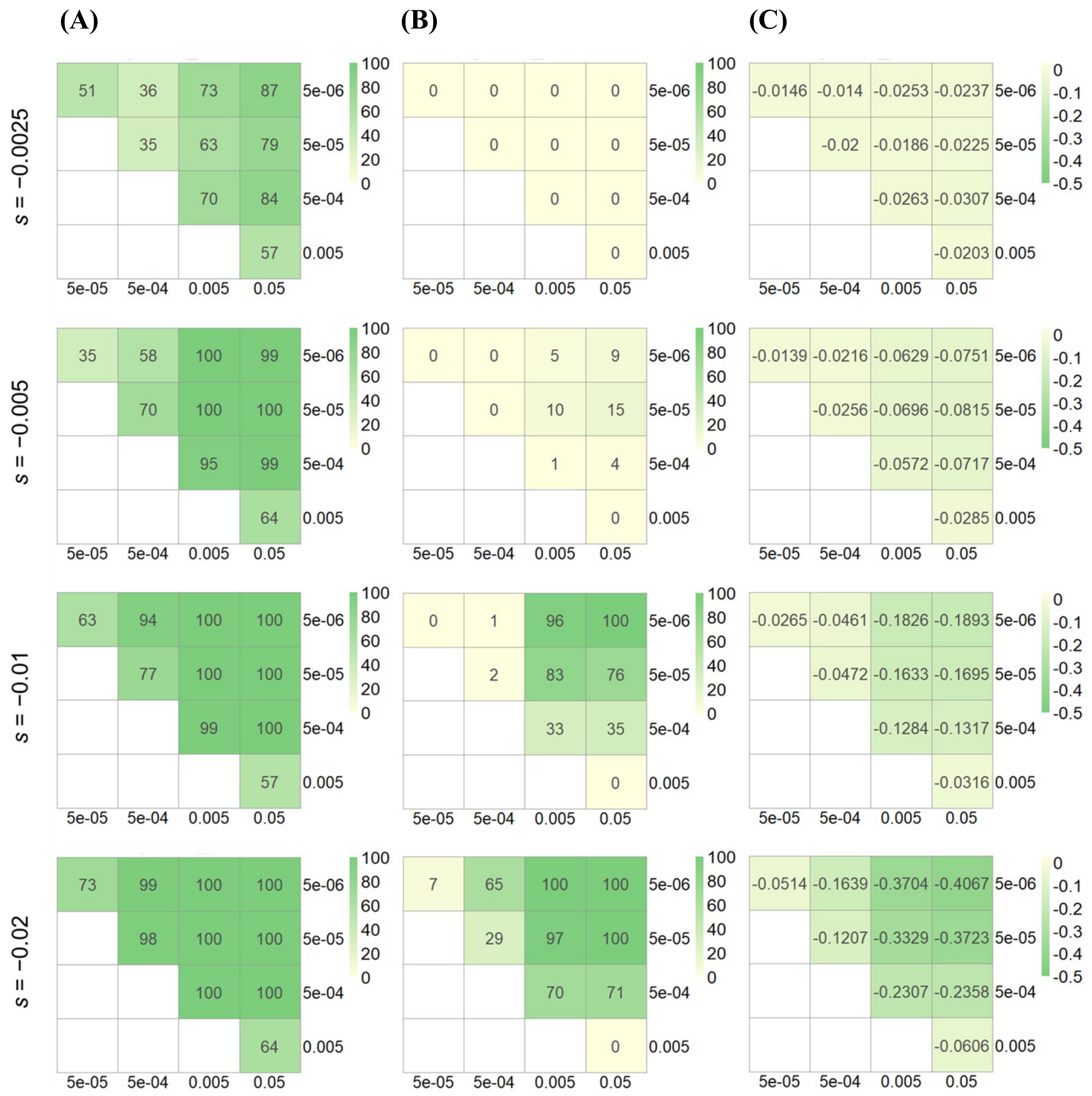

Figure S5. Simulations showed the power of the pMK test varied with migration rates and selection coefficients (negative selection). The power of the pMK test across different levels of divergence time was examined through simulating various migration rates. Higher migration rates represent lower divergence times. **(A)** The number in each box indicates the number of replicates showing the expected pattern of positive selection (i.e., A/B < C/D) out of 100 replicates. The majority of replicates showed expected patterns, and the consistency was the

lowest when migration rates were both high or the strength of selection was weak ($s = -0.0025$). **(B)** The number in each box indicates the number of replicates with significant pMK test results (p-value <0.05) out of 100 replicates. The number of significant replicates increased with the difference in migration rates and the selection coefficient. **(C)** The number in each box represents the average log odds ratio (i.e., $\log\left(\frac{A/B}{C/D}\right)$). The absolute value of the average log odds ratio increased with the selection coefficient and the difference in migration rates.

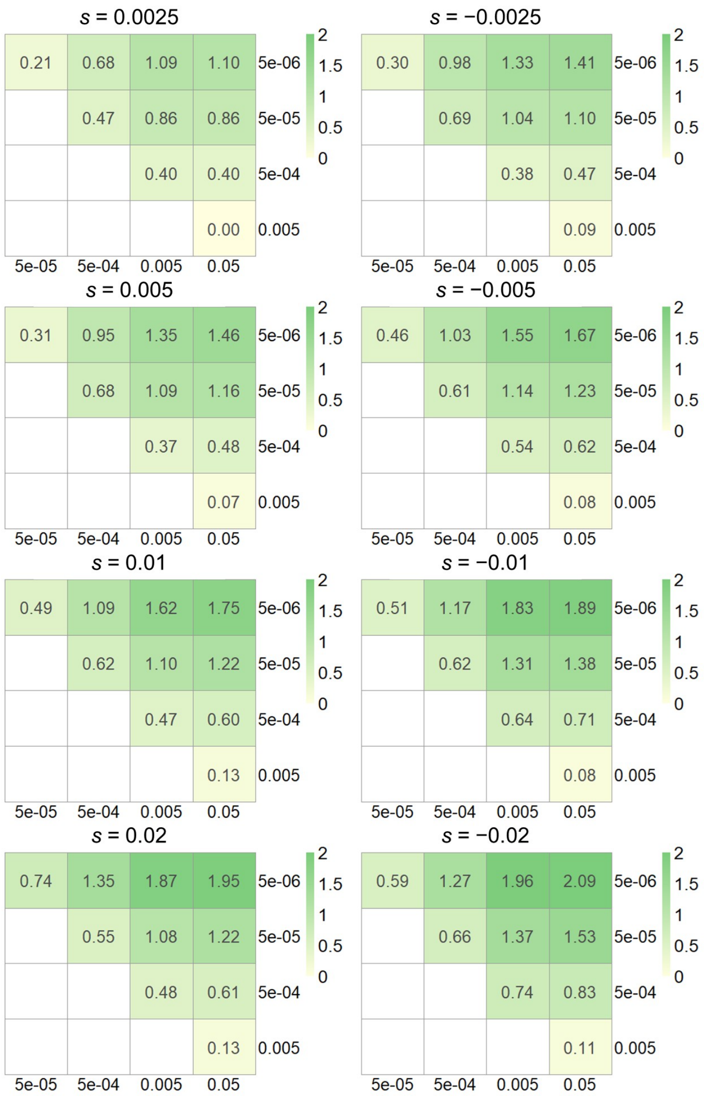

Figure S6. The log *S* ratio under various migration rates and selection coefficients. The *S* ratio was influenced by the difference in migration rates and the selection coefficient. While the *S* ratio was calculated from the number of neutral changes, the evolutionary dynamics of neutral mutations was influenced by deleterious or beneficial mutations on the same genome.

Table S1. The domains of the envelope protein and NS1

Envelope			
Serotype	Domain 1	Domain 2	Domain 3
1	1-51, 133-191, 275-298	52-132, 192-274	299-396
2	1-51, 132-192, 280-295	52-131, 193-279	296-393
3	1-51, 132-190, 278-293	52-131, 191-277	294-391

NS1					
Serotype	Domain 1	Domain 2	Domain 3	Domain 4	Domain 5
1, 2, 3	21-40	101-135	156-175	231-255	296-335

References

1. Katoh K, Rozewicki J, Yamada KD. 2019. Mafft online service: Multiple sequence alignment, interactive sequence choice and visualization. Brief Bioinform. 20(4):1160-1166.
2. Kuraku S, Zmasek CM, Nishimura O, Katoh K. 2013. Aleaves facilitates on-demand exploration of metazoan gene family trees on mafft sequence alignment server with enhanced interactivity. Nucleic Acids Res. 41(Web Server issue):W22-28.
3. Martin DP, Murrell B, Golden M, Khoosal A, Muhire B. 2015. Rdp4: Detection and analysis of recombination patterns in virus genomes. Virus Evol. 1(1):vev003.
